# Global survey of malaria rapid diagnostic test (RDT) sales, procurement and lot verification practices: assessing the use of the WHO–FIND Malaria RDT Evaluation Programme (2011–2014)

**DOI:** 10.1186/s12936-017-1850-8

**Published:** 2017-05-15

**Authors:** Sandra Incardona, Elisa Serra-Casas, Nora Champouillon, Christian Nsanzabana, Jane Cunningham, Iveth J. González

**Affiliations:** 1 FIND, Geneva, Switzerland; 2 Independent Consultant, FIND, Geneva, Switzerland; 3 Global Malaria Programme, World Health Organization (WHO-GMP), Geneva, Switzerland

**Keywords:** Malaria, Rapid diagnostic test, Quality control, Product testing, Lot testing

## Abstract

**Background:**

Malaria rapid diagnostic tests (RDTs) play a critical role in malaria case management, and assurance of quality is a key factor to promote good adherence to test results. Since 2007, the World Health Organization (WHO) and the Foundation for Innovative New Diagnostics (FIND) have coordinated a Malaria RDT Evaluation Programme, comprising a pre-purchase performance evaluation (product testing, PT) and a pre-distribution quality control of lots (lot testing, LT), the former being the basis of WHO recommendations for RDT procurement. Comprehensive information on malaria RDTs sold worldwide based on manufacturers’ data and linked to independent performance data is currently not available, and detailed knowledge of procurement practices remains limited.

**Methods:**

The use of the PT/LT Programme results as well as procurement and lot verification practices were assessed through a large-scale survey, gathering product-specific RDT sales and procurement data (2011–14 period) from a total of 32 manufacturers, 12 procurers and 68 National Malaria Control Programmes (NMCPs).

**Results:**

Manufacturers’ reports showed that RDT sales had more than doubled over the four years, and confirmed a trend towards increased compliance with the WHO procurement criteria (from 83% in 2011 to 93% in 2014). Country-level reports indicated that 74% of NMCPs procured only ‘WHO-compliant’ RDT products, although procurers’ transactions datasets revealed a surprisingly frequent overlap of different products and even product types (e.g*., Plasmodium falciparum*-only and *Plasmodium*-pan) in the same year and country (60 and 46% of countries, respectively). Importantly, the proportion of ‘non-complying’ (i.e., PT low scored or not evaluated) products was found to be higher in the private health care sector than in the public sector (32% vs 5%), and increasing over time (from 22% of private sector sales in 2011 to 39% in 2014). An estimated 70% of the RDT market was covered by the LT programme. The opinion about the PT/LT Programmes was positive overall, and quality of RDTs as per the PT Programme was rated as the number one procurement criteria.

**Conclusions:**

This survey provided in-depth information on RDT sales and procurement dynamics, including the largely unstudied private sector, and demonstrated how the WHO–FIND Programme has positively influenced procurement practices in the public sector.

**Electronic supplementary material:**

The online version of this article (doi:10.1186/s12936-017-1850-8) contains supplementary material, which is available to authorized users.

## Background

Rapid diagnostic tests for malaria (RDTs) play a critical role in malaria case management, especially in remote areas of malaria-endemic countries, where good-quality microscopy is not always available. Since their emergence on the market in the early 1990s, the number of malaria RDT brands has substantially increased [1]. However, malaria RDTs from different manufacturers can present wide variations, especially in terms of performance characteristics, and can be affected by sub-optimal transport or storage conditions [2, 3].

The World Health Organization (WHO) and the Foundation for Innovative New Diagnostics (FIND) coordinate a Malaria RDT Evaluation Programme aimed at monitoring the quality of RDTs along the supply chain [4]. The Programme comprises pre-purchase performance evaluation (product testing, PT) and post-purchase quality control (lot testing, LT). The PT programme compares commercially available RDTs and publishes comparative data on performance, ease of use and stability, with 247 products having been evaluated as of 2015 [5–11]. This programme currently constitutes the basis of WHO procurement recommendations [12], and forms the laboratory evaluation component of the WHO Prequalification of Diagnostics Programme (WHO PQ). To achieve prequalification status, malaria RDTs not only need to fulfil the WHO recommended quality standards in the PT programme, but also have to successfully undergo a product dossier review and an inspection of the manufacturer’s site(s) by the WHO PQ Programme. Prequalification will become a requirement for WHO procurement recommendation at the end of 2017. The lot testing (LT) programme evaluates samples of individual product batches (or ‘lots’) before distribution and use, thereby stimulating manufacturers to maintain RDTs at a consistently high-quality standard, but also increasing the confidence of RDT procurers and users. This LT service can be requested by any entity that would like to confirm the quality of RDT lots, i.e. the RDT manufacturers or various types of RDT procurers such as Ministries of Health (MoH), National Malaria Control Programmes (NMCP), non-governmental organizations (NGO), procurement agencies and other international organizations (IO); RDT procurers can also ask the RDT manufacturers to handle the lot testing request as part of the procurement contract. Since 2007, more than 4000 malaria RDT lots have been tested at two WHO–FIND-recognized Lot Testing laboratories, based in The Philippines and Cambodia [13].

There has been a noticeable increase of malaria RDT sales worldwide (i.e, from 50 million in 2008, to 314 million in 2014 [14]), and market trends have been documented in some publicly available reports. UNITAID’s annual Malaria Diagnostics Technology and Market Landscape report [15–17] describes the malaria RDT market in terms of demand, pricing and manufacturer share, and the WHO Global Malaria Programme reports yearly updates of the total RDT sales from the main manufacturers [14]. However, neither of these reports publishes details on product-specific dynamics or country-specific procurement practices. In 2011, a survey conducted by FIND on 17 manufacturers provided annual figures on the estimated proportion of high-quality products reaching the market, demonstrating a general shift to better-performing malaria RDTs [18]; however, there has been no follow-up assessment of product-specific RDT sales since then. In addition, there is a critical knowledge gap around sales of RDTs in the private health care sector, where 40–60% of people seek care for febrile illnesses in some endemic countries [19]. A snap-shot of RDT availability and quality in private health care outlets of six countries was published by Albertini et al. [20]. However complete and reliable data on overall procurement practices and RDT performance in this sector are not available. Similarly, estimates on the overall market coverage of the LT programme have been difficult to make due to lack of compiled data on quantities of lots released or the sizes of production lots.

The survey presented herein was commissioned by FIND to better document the use of PT and LT programme results to inform procurement and lot verification practices. The main objectives were to gather product-specific information of world-wide malaria RDT sales to public and private sectors, assess the use of PT results for the selection of RDT products, and to accurately estimate the coverage of the LT programme with reference to real lot-manufacturing output data. Additionally, detailed information on RDT procurement and manufacturing practices was gathered in order to facilitate interpretation of RDT market observations.

## Methods

### Survey target contacts

The survey comprised three different groups:

#### National Malaria Control Programmes/Ministries of Health

All malaria-endemic countries for which country profile information was available in World Malaria Report [21] were contacted for this survey.

#### Malaria RDT procurers

This group, referred to by the general term ‘procurers’, includes different types of organizations involved in malaria RDT purchasing (procurement agents, funding agencies, IOs and NGOs), acting either on a global or local context. ‘Global procurers’ included the major funding agencies [The Global Fund to Fight AIDS, Tuberculosis and Malaria (Global Fund), the US President’s Malaria Initiative (US-PMI), World Bank] and major international RDT institutional buyers such as the Partnership for Supply Chain Management (PFSCM) in charge of the Global Fund Voluntary Pooled Procurement (VPP), John Snow International (JSI), United Nations Children’s Fund (UNICEF) or Médecins sans Frontières (MSF), among others. ‘Local procurers’ included country-based or regional offices of organizations involved in RDT procurement that had previously submitted malaria RDT lots to the LT programme (as per FIND’s LT database).

#### Malaria RDT manufacturers

All companies that had participated in at least one of the PT rounds (Rounds 1 to 6) [5–10] were contacted for this survey.

### Survey procedure and requested data

The survey was based on questionnaires sent by e-mail with subsequent follow-up by phone calls as required. Questionnaires included 10–13 predefined queries (Table 1, Additional files 1, 2 and 3) and were accompanied by a description of the survey objectives and some background about the PT and LT programmes. The NMCP/MoH questionnaires were made available in French and Spanish to francophone and hispanophone country contacts, respectively, and Portuguese versions were offered to lusophone countries upon request. The survey started in mid-November 2014, and was completed in March 2015. All communications included a confidentiality statement, hence no product-specific nor company/organization-specific information can be made publicly available.

**Table 1:** Summary of data requested in the questionnaires for each survey category

NMCP/MoHs	Procurers	Manufacturers
Awareness, use and opinion of the PT and LT programmes		
RDT procurement practices (e.g., product selection criteria, funding source)		RDT manufacturing practices (e.g., lot-sizing, internal quality control procedures)
RDT procured products (2011–2014 period)		RDT sold products (2011–2014 period)
Product code Year	Product code Year Number of tests Country	Product code Year Number of tests Number of lots% sales destined to public and private sector

NMCP National Malaria Control Programme, MoH Ministry of Health, PT product testing, LT lot testing, RDT rapid diagnostic test

### Additional data sources used for the analysis

The sales information gathered from RDT manufacturers, i.e. number of RDTs sold per each product code (or catalog number), and procurement information gathered from RDT procurers and NMCPs/MoHs, i.e. number of RDTs procured for each given RDT product code, were compared with the WHO–FIND data of the PT and LT programmes, to determine whether (1) sold/procured malaria RDTs fulfilled the quality requirements of the WHO RDT procurement recommendations, and (2) if they had been lot-tested before purchase and distribution. This analysis obliged using the following additional data sources:

#### Results of the PT evaluations

All product data entries (sales or procurement) communicated by the survey respondents were assigned to one of three categories, specifically, COMPLY/NOT COMPLY/NOT TESTED based on the PT performance of the product on the year of the transaction [5, 6, 8, 9], i.e. it was verified if the product sold or procured did meet or not the quality requirements listed in the WHO recommendations for procurement of RDTs [1, 12, 22, 23]; exact definitions are detailed further below. For the purpose of this study, the outcomes of the first four rounds of PT were taken into account (Round 5 results have not been included having only been published in July 2014 given there is always a delay before RDT procurers start using updated PT results).

#### Results of the LT evaluations

The number of lots evaluated per each product (identified by product code) and year was extracted from the LT Programme’s database. Duplicated evaluations of the same lot (e.g., submitted by different requesters, pre- and post- shipment, etc.) were excluded for the computation. For the estimation of the LT coverage, the number of lots submitted to LT was compared to the number of lots sold (as reported by the manufacturers). The available information on annual product-specific lot sizes allowed for estimates of the number of RDTs covered by LT.

#### The global fund price and quality reporting (PQR) system (downloaded on 5 May 2015 [24])

This publicly available database compiles information on purchases of health products executed with Global Fund resources. Procurement transaction details are entered by the grant recipients upon receipt of goods. As a consequence, this dataset presents some limitations, i.e., not totally complete, reporting delay, potential errors in data entry. However, the bulk of information contained in this database makes it still one of the most valuable sources of data for RDT procurement worldwide transactions.

### Analysis and definitions

Qualitative data (i.e., text replies with suggestions and opinions) was classified into different categories of main comments (even if expressed in different ways). Detailed information provided through these replies cannot be disclosed due to its confidential nature. Quantitative data are provided in the form of aggregated figures as well, to preserve confidentiality. Country results are represented according to WHO region categories: Regional Office for Africa (AFRO), the Americas (AMRO), Europe (EURO), the Eastern Mediterranean (EMRO), Southeast Asia (SEARO), and the Western Pacific (WPRO). Manual data cross-checks were conducted to verify or complete information and to discard redundant entries received from different sources (e.g., PQR and IO direct reports). For compliance of RDT products with WHO procurement recommendations, products had to fulfill the following requirements: Panel Detection Score (PDS) for *Plasmodium falciparum* detection being ≥50% (for 2011 data) or ≥75% (for 2012–14 data), PDS for *Plasmodium vivax* detection (if applicable; i.e., combination tests) ≥75% for 2011–14 data, false positive rates <10%, invalid rates <5%. PT reports used as the reference were: Rounds 1–2 for 2011 data, Rounds 1–3 for 2012 data, and Rounds 1–4 for 2013–14 data. A few considerations should be taken into account: (a) sales data from manufacturers that replied before end of 2014 and did not provide full-year records for the last year were extrapolated by a prorated calculation based on available data, with corrections accounting for a 1% increase of the overall registered sales for 2014; (b) for the assignment of procurement transactions to a particular year, it should be noted that the datasets received from different procurers were not fully standardized, i.e., referring to different time points of the procurement process (date of order, date of shipment, date of delivery, etc.); (c) procurement transactions corresponding to different pack sizes (e.g., kits of 25, 30 or 50 RDTs of the same product) or formats (e.g., kits with single-use packs of RDTs) of a same product were grouped under a lumped product code; (d) for estimation of sales going to the public vs the private health care sector, manufacturers were asked to provide an estimate of their sales going to the public sector on an annual basis, with a single estimate applicable to all products of the company (to facilitate reporting), and the remainder was estimated to go to the private sector. Product-specific analyses were done by using this estimate for all product codes hence not accounting for possible differences in the sales to the public vs the private sector between products of the same company; (e) for the purpose of this study, the public sector was considered to refer to institutions procuring RDTs for a not-for-profit purpose, i.e. public institutions (e.g. Ministries of Health, National Malaria Programs, etc.), international organizations (e.g. UNICEF, UNDP, etc.), international NGOs (e.g. MSF) and major funding/procurement agencies (e.g. Global Fund-PFSCM, USAID-JSI, etc.) managing large-scale orders aimed at public sector supply. This definition would therefore exclude any orders destined to the private for-profit sector, e.g. private clinics, private medical device distributors, pharmacies or drug stores; and, (f) for one manufacturer, the annual number of lots evaluated by LT slightly exceeded the reported quantities of lots sold per year, probably because of possible time shifts between sales and lot testing. In this particular case, the estimations of RDT volumes covered by LT were adjusted to avoid over-reporting.

## Results

### Survey participants

#### NMCP/MoH survey data

Overall, 100 countries were contacted and replies were received from 79, including 11 who reported not to be using malaria RDTs (six AMRO, two EURO, one EMRO, one WPRO, one AFRO). Therefore, a total of 68 (68%) countries were included in the analysis. Three (4%) out of the 68 did not fully reply to one of the queries; however information on procured RDT products was provided by all 68 respondents. More than half of the NMCP/MoHs participating in the survey belonged to the AFRO region (38/68, 56%). Questionnaire respondents were mostly NMCP coordinators or country focal points for malaria diagnostics. Contributions from Central Medical Stores or National Laboratories (MoH) were also reported in a few questionnaires.

#### Procurers survey data

Eighteen out of 25 procurers (72%) responded to the questionnaire, with a higher response rate among Global procurers (13/14, 93%) than Local procurers (5/11, 45%). Six contacts were not directly conducting procurement, i.e. they were only operating as funding agencies or not currently purchasing malaria RDTs, which results in a final response rate of 48% (12/25). One (8%) out of the 12 respondents did not provide a fully completed questionnaire. As for product data, nine (75%) of the 12 participants provided a duly-completed procurement transaction file for the 2011–14 period. Survey respondents were mostly procurement specialists/managers or quality assurance officers. Pharmacists and supply-chain coordinators also contributed for some organizations.

#### Manufacturers survey data

Forty-two out of 62 (68%) manufacturers responded to the questionnaire, including one who expressed concerns about sharing confidential data, and four considering that the survey was not applicable for their companies (e.g., not currently producing malaria RDTs). Therefore, a total of 37 (60%) questionnaires were returned and included in the analysis. Sales data were made available by 32 (86%) of the respondents (three companies had not sold RDTs, and two indicated confidentiality reasons), while the rest of questionnaire replies were fully completed by all participants except one (3%) that stated insufficient experience with the Programme (i.e., had just recently submitted a product for the first time). Among the respondents, 65% were companies based in Asia, 16% in North America and 11% in Europe (the remaining 8% corresponds to one Australian and two South African manufacturers). More than half of the replies were sent by the chief executive officer or product manager of a company. Feedback from the Regulatory Affairs or Quality Assurance departments was also common, whereas direct replies from Overseas Sales and Marketing departments were only received in a few cases.

### Malaria RDT procurement

#### NMCP/MoHs

A total of 157 malaria RDT procurement transactions for the 2011–14 period were reported by the 68 NMCP/MoHs included in the analysis. Overall, country respondents reported 31 different product codes, although there were four main products that accounted for more than half (53%) of the procurement transactions. For the whole survey period, each NMCP/MoH reported an average of 2.3 product codes (range 1–6), and overlapping of different products and product types (e.g., Pf-only, Pan/Pf) during the same year was found in 46 and 19% of the countries, respectively. The main sources of funding cited by NMCP/MoHs were the Global Fund and government funding (84 and 63% of reported cases, respectively), followed by other funding sources such as US-PMI, UNICEF and the World Bank (34, 21, and 15% respectively). The government was largely responsible for the procurement process (e.g., Central Medical Store, Pharmacy department, NMCP, etc.) in 53% of the cases, and a procurement or funding agency such as the Global Fund, US-PMI or UNICEF conducted the procurement in 36% of the cases. When asking the NMCP/MoH contacts about knowledge about other organizations procuring RDTs in their country, nearly half of them (46%) cited examples such as NGOs, private distributors or international organizations.

#### Procurers

After pooling the data received from the nine responding procurers, a total of 1065 transactions for the 2011–14 period (representing 622,618,199 RDT units) were included in the analysis. Overall, 21% of these RDTs were directly purchased by principal recipients of funding agencies (i.e., direct purchase to the manufacturer by country-based grant administrators), whereas the rest were mainly procured through procurement agents, major institutional buyers or IOs/NGOs central offices. The malaria RDTs reported in this survey group were distributed in 78 different countries, with ten African countries accumulating the 60% of the worldwide reported volumes. For the whole survey period, an average of 3.3 product codes (range 1–9) per destination country was reported. Overlapping of different product codes in the same year was observed in 60% of the countries (up to six different products in circulation), whereas different product types (e.g., *Plasmodium falciparum*-only, Pan/*P. falciparum*, etc.) were found to overlap during the same year in 46% of the countries. The presence of simultaneous procurers (from two to six) distributing RDTs in a specific country and year was detected in 61% of the countries.

### Manufacturer malaria RDT sales

**Fig. 1:**
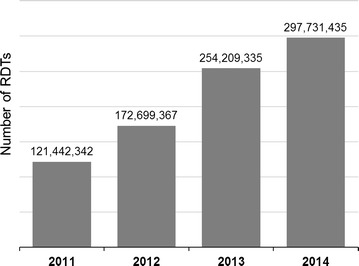
Number of malaria RDT units sold annually (2011–14) as reported by RDT manufacturers. Sales data provided by 32 malaria RDT manufacturers; 106 RDT products reported

Sales data received from 32 manufacturers included 106 malaria RDT products, with an average number of products (i.e., product codes) reported per manufacturer of 3.31 (range 1–9), and a total sales volume of 846,082,479 RDT units, corresponding to 6295 lots, for the 2011–14 period. The total amount of malaria RDTs released in the market more than doubled over the 4 years (Fig. 1). According to manufacturers’ reported estimations, malaria RDT sales were mainly destined to the public sector (84%), with a market growth over the 2011–14 period being higher for public sector sales (2.6-fold raise) than for the private sector sales (1.8-fold raise). Eleven out of 32 manufacturers were exclusively selling RDTs to the private sector, and another seven reported that more than half of their sales were going to that sector.

### Performance of procured RDTs according to PT results

#### Based on NMCPs/MoH procurement reports (2011–14)

Within the list of 31 product codes reported by country respondents for the 2011–14 period, 19 met the WHO procurement criteria (61%), six products did not, and six were products not having been evaluated by the PT programme. However, overall, 136 (87%) of the NMCP-reported transactions corresponded to ‘complying’ RDT products, whereas ‘non-complying’ and ‘non-tested’ products were reported in 15 (10%) and six (4%) transactions, respectively.

The compliance of NMCPs/MoH with WHO criteria for RDT procurement was evaluated based on a country performance grade. Briefly, NMCP/MoHs were classified as ‘high-compliers’ when all procured products complied with the recommendations, ‘medium-compliers’ when some products complied but others did not, or were not tested, and ‘low-compliers’ when all procured products did not comply or were not evaluated by the PT programme.

**Fig. 2:**
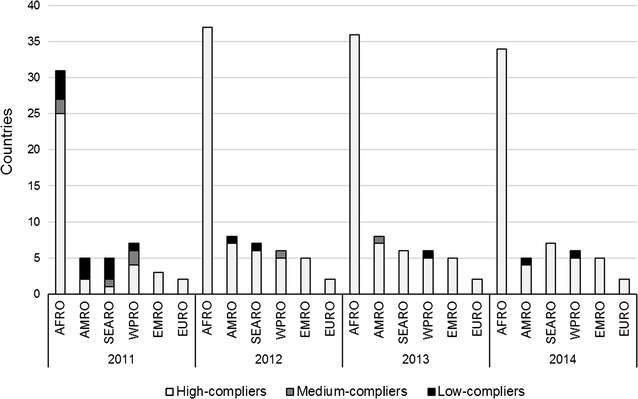
Annual compliance of NMCP/MoHs with the WHO criteria for RDT procurement, per region (2011–14). Number of countries in each WHO region and compliance category, per year. Country performance was graded as ‘high-compliers’ (all procured products complied with recommendations), ‘medium-compliers’ (some products complied but others did not, or were not tested) and ‘low-compliers’ (all products did not comply or were not evaluated by the PT programme). NMCP/MoH National Malaria Control Programme/Ministry of Health, AFRO WHO Regional Office for Africa, AMRO WHO Regional Office for the Americas, EURO WHO Regional Office for Europe, EMRO WHO Regional Office for the Eastern Mediterranean, SEARO WHO Regional Office for Southeast Asia, WPRO WHO Regional Office for the Western Pacific

For the whole 2011–14 period, a total of 50 (74%) countries were categorized as ‘high-compliers’, whereas 18 (26%) showed ‘medium compliance’. Countries from AMRO, SEARO and WPRO were more frequently rated as medium-compliers (5/8, 4/7 and 3/7, respectively) compared to the NMCP/MoHs from the AFRO region (6/38). The annual breakdown of these data shows that most cases of ‘medium’ or ‘low compliance’ were observed in 2011, but then decreased, with only one AMRO and one WPRO country being classified as ‘medium-’ or ‘low-complier’ in the two last years of the survey (Fig. 2). None of the EMRO and EURO countries participating in this survey (n = 8) reported products that did not comply with WHO recommendations.

**Fig. 3:**
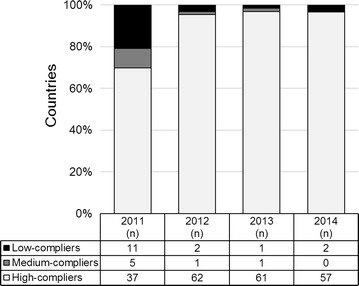
Annual compliance of NMCP/MoHs with the WHO criteria for RDT procurement (2011–14). Percentage (graph) and number (Table) of countries. Country performance was graded as ‘high-compliers’ (all procured products complied with recommendations), ‘medium-compliers’ (some products complied but others did not, or were not tested) and ‘low-compliers’ (all products did not comply or were not evaluated by the PT programme). NMCP/MoH: National Malaria Control Programme/Ministry of Health

Countries procuring malaria RDTs with exclusive government funding (n = 8) were more frequently rated as ‘medium-compliers’ (5/8) compared to NMCP/MoHs receiving total or partial funding from external donors (13/60); all countries reporting exclusive self-funding and rated as ‘medium-compliers’ (n = 5) were from AMRO, SEARO or WPRO regions. When the NMCP/MoH performance analysis was done on an annual basis, a noticeable proportion of ‘medium-’ and ‘low-compliers’ was found in 2011 (9 and 21%, respectively), but a clear improvement of the compliance was observed over the four years (from 70% ‘high-compliers’ in 2011, to 97% in 2014) (Fig. 3).

#### Based on procurers’ reports (2011–14)

**Fig. 4:**
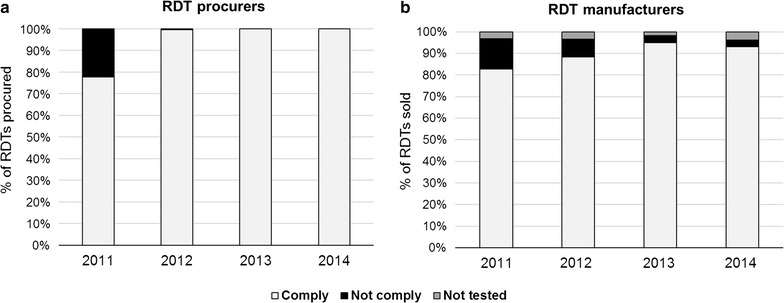
Annual compliance of procured/sold malaria RDT products with the WHO criteria for RDT procurement (2011–14).** a** Percentage of RDTs procured per year, as reported by RDT procurers (e.g., funding agencies, procurement agents, NGOs, IOs);** b** percentage of RDTs sold per year. Compliance categories are based on the PT performance of the RDT product on the year of the transaction

Among the total amount of procured RDTs reported for the whole survey period (i.e., 622.6 million RDTs), 17.2 million RDTs (3%) were not complying with the WHO criteria. As shown in Fig. 4a, most of the transactions involving procurement of non-complying products occurred during 2011 (16.4 million RDTs). More than half (54%) of the products that did not follow the WHO recommendations were directly procured by principal recipients of large funding agencies.

#### Based on manufacturers’ sales reports (2011–14)

Among the total sales transactions reported for the survey period (i.e., 846.1 million RDTs), 49.7 million RDTs (6%) were not complying with WHO criteria and 25.1 million RDTs (3%) had not been evaluated by the PT programme. Between 2011 and 2014, the percentage of sold products complying with WHO criteria showed an increase from 83 to 93%, whereas the sales percentage of non-complying products decreased from 14 to 3% (Fig. 4b).

### Comparison of sales to the public vs the private health care sector

**Fig. 5:**
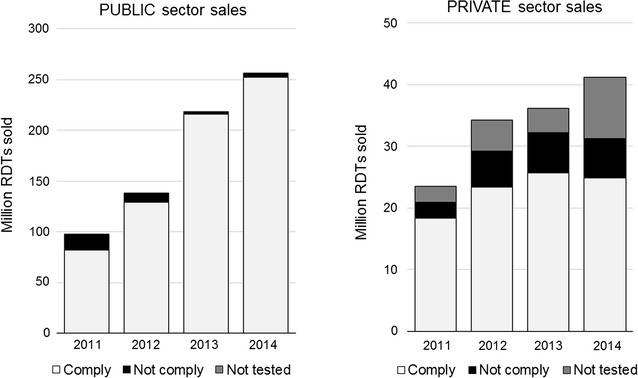
RDT sales in the public vs the private health care sector: annual compliance with the WHO criteria for procurement (2011–14). Number of RDTs delivered in the market. Compliance categories are based on the PT performance of the RDT product on the year of the transaction

The RDT volumes reported for each product were split according to the estimated annual ratios of sales to the public sector provided by each manufacturer. For the whole survey period, the proportion of products that did not comply with WHO criteria or had not been evaluated was found to be higher within the private sector (32%) compared to the public sector (5%). Importantly, private sales accounted only for small fraction of the total reported transactions of this survey (around 16%), however, the absolute number of RDTs that did not meet WHO recommendations was still higher in the private sector sales (43 million RDTs) compared to the total public sector sales of non-compliant RDTs (32 million RDTs). A clear trend towards increased WHO compliance was observed for the public sector sales (from 84% in 2011, to 93%, 99% and then 98% in 2012, 2013 and then 2014, respectively) (Fig. 5) but, conversely, the percentage of products complying with the WHO recommendations was gradually decreasing in the private sector (from 78% in 2011, to 68%, 71% and then 61% in 2012, 2013 and then 2014, respectively).

The manufacturers’ reported presence in the public vs the private sector was found to differ according to the compliance of their marketed products with WHO criteria. For the 2011–14 period, between eight and 13 manufacturers were annually categorized as distributors of ‘high-scoring’ products (i.e., reporting higher sales volumes of complying RDTs), whereas 14–20 manufacturers were categorized as distributors of ‘low-scoring’ products (i.e., delivering greater volumes of non-complying or not-tested RDTs). Distributors of ‘high-scoring’ products were those reporting a wider presence in the public sector (63.9%) as well as accounting for the biggest proportion of market share (790 million RDTs; 93% sales), compared to distributors of ‘low-scoring’ products, with only 19.1% average sales destined to the public sector and also smaller market share (56 million RDTs; 7% sales).

### Lot testing coverage

During the 2011–14 period, 2510 lots corresponding to 30 different products from 12 manufacturers were evaluated by the LT programme. Among them, three products had not been reported by the manufacturers participating in the survey, so the corresponding LT episodes (i.e., lot testing events of these particular products, n = 6) were not included in this analysis. Overall, 40% (2504/6295) of the total number of reported lot sales was submitted to the LT programme (Table 2). The ten product codes with higher sales volumes had more than 70% of their lots evaluated, with only two exceptions for which 66 and 44% of lots were tested. The available information on number of lots and lot sizes was used to estimate LT coverage of the RDT sales volumes, showing that an estimated 70% of the global market is covered by the LT Programme (Table 2). The amount of products covered by the programme showed an increase, from 69 million RDTs covered in 2011, to 211 million RDTs in 2014.

**Table 2:** Annual coverage of the lot testing programme (referred to reported RDT sales)

	Evaluated by lot testing^a^	Delivered (reported sales)^b^	Percentage of market coverage^c^ (%)
2011			
Lots	334	1310	25
RDTs	68,842,861	121,442,342	57
2012			
Lots	480	1525	31
RDTs	105,454,077	172,699,367	61
2013			
Lots	908	1708	53
RDTs	208,914,128	254,209,335	82
2014			
Lots	782	1752	45
RDTs	211,334,091	297,731,435	71
(2011–14)			
Lots	2504	6295	40
RDTs	594,545,158	846,082,479	70

^a^Number of lots or RDTs that underwent the WHO-FIND malaria RDT lot testing, as per the lot testing database

^b^Number of lots or RDTs sold as per the survey data gathered from the RDT manufacturers

^c^Coverage of the Lot Testing programme, estimated by calculating the percentage of lots or RDTs that have been lot tested, relative to the total number of lots or RDTs sold in a given year

### Awareness, opinions and suggestions about the PT/LT programmes

The reported awareness on the PT and LT programmes was high among procurers (91 and 100%, respectively) and slightly less among the NMCP/MoHs (82 and 85%, respectively). Regarding the interactive guide for malaria RDT selection [11], replies indicated that it is not yet a widely used tool (a 38% of countries and 42% of procurers do not use it or know about it). NMCP/MoHs were often not informed about the requirements set by their donors to select RDTs (i.e., more than 75% of the respondents that did not know about preconditions related to PT/LT programmes (n = 34) were buying products through major funding agencies/IOs that strictly follow WHO procurement criteria). Only one-third of countries acknowledged mentioning the WHO–FIND malaria RDT evaluation in their national official documents (e.g., country guidelines for malaria diagnostic, technical specifications for RDT procurement), whereas 75% (8/12) of the procurers refer to the WHO–FIND programmes in their Quality Assurance guidelines or tender documents.

Most survey participants (96/106; 91%) had a positive opinion of WHO–FIND malaria RDT Evaluation programmes (all mean scores fell between 7 and 8, on a 0–10 scale). Twelve (22%) out of the 55 NMCP/MoHs who provided comments explicitly pointed out the Programme’s usefulness to guide country decision-making. Manufacturers highlighted the contribution to the enhancement of customers’ confidence, as well as the impact that the PT programme had on the quality of their products, with 15 (42%) manufacturer respondents recognizing that it had triggered changes in their manufacturing and quality control procedures. Suggestions for improvement depended on the survey group. NMCP/MoHs were interested in a decentralization of the Programme and set up of country-based evaluation laboratories, while manufacturers’ recurrent demand was to increase availability of positive controls for manufacturing and quality control purposes. Procurers advocated random sampling of tested RDTs, as well as an increased and improved diffusion of the Programme results (e.g., share lot-numbers, publish results of accessories’ evaluations, facilitate report interpretation).

## Discussion

This large-scale survey allowed assessment of the influence of both the PT and LT programmes on procurement and quality control practices, based on first-hand sales and procurement data. Considering that this survey was based on voluntary participation, the questionnaire response rates achieved for NMCP/MoHs (68%), manufacturers (60%) and procurers (48%) were perceived as satisfactory. In addition, a number of non-responding contacts argued they were not applicable targets (e.g., countries not procuring RDTs, manufacturers not currently producing malaria RDTs).

### Volumes and types of RDTs being sold and procured worldwide

The global sales volumes compiled through the manufacturers’ survey (from 121 million RDTs in 2011, to 298 million in 2014) showed the same upward annual trend as reported in the WHO World Malaria Report (from 155 million RDTs in 2011, to 314 million in 2014), with the only difference that the sales reported here indicated a continued increase up to 2014 instead of a trend to stabilize after 2013 (i.e., 319 million RDTs reported by WHO in 2013) [14]. Market volumes compiled by WHO were moderately higher (i.e., 5–28% above sales reported in the present survey) although the number of surveyed manufacturers was slightly lower (24–29 vs 32, respectively). One reason might be that manufacturers did not report a small part of their sales because the detailed information requested in this survey (e.g., product-specific data, number of lots) was not available or not included for other unknown reasons in their datasets (e.g., some missing product codes were noted). Of note, the full sales data collected through these surveys can still be an underestimation (i.e., missing manufacturers that do not participate in PT and/or did not reply to the survey requests).

The malaria RDT procurement trends inferred from the survey data were also in accordance with the outcomes reported in the UNITAID Malaria Diagnostics Landscape reports [15–17]. Malaria RDT procurement records revealed a frequent co-existence of multiple RDT products in country, occurring in 46 and 60% of countries according to the NMCP/MoH and Procurers data, respectively, with up to six different product codes reported in the same year and country. Even more surprising was the overlapping of different product types (e.g., *P. falciparum*-only, Pan/*P. falciparum*) in the same year, reported for 19 and 46% of countries according to NMCP/MoHs and procurers’ datasets, respectively. This was unexpected since different product types require a different results interpretation because of the presence of different types of test lines, hence health care staff needs to be trained accordingly to avoid confusions. Survey data also confirmed that in almost two-thirds of the countries there was more than one organization involved in RDT procurement. Overall, these results indicate that the different players of RDT procurement at national level do not necessarily coordinate or aim to procure the same product for the country. Importantly, unless training programmes and supervision are well coordinated, concurrent use of different RDT products can seriously increase the risk of performance errors, especially among less-skilled malaria RDT end-users [25, 26].

### Compliance of RDTs with WHO procurement criteria

Overall, compliance with WHO recommendations for selection of malaria RDT has been improving since 2011. The shift to better-performing products was observed in the three survey groups, and can be explained by multiple factors. First, the increased awareness of the programme among malaria RDT procurers and country decision-makers has prompted changes on their selection and purchasing criteria, including modifications of the procurement policies by all major RDT funding agencies since 2010. This survey also shows that around 75% of RDT procurers include programme-based conditions in their tender documents or guidelines. Overall, this has probably contributed to the reduction of poor-performing products in the RDT market. More specifically, several product-specific sales fluctuations detected in the market analysis (not disclosed) were closely associated with updated product results in PT evaluations. Second, PT has stimulated manufacturers to improve the performance of their products. In this survey, almost half of the malaria RDT manufacturers acknowledged the introduction of manufacturing improvements triggered by the WHO–FIND programme, such as modifications on their lot-release procedures or internal quality control panels. In line with this, the great majority of products that were re-submitted to PT evaluation either improved or maintained their high score, and several products were discontinued after consecutive low PT scoring [5–10]. A product-specific sales analysis of the survey data (not disclosed here) also indicates a clear increase in the relative market share of a few ‘popular’ products which have been consolidated during the last years as good PT performers and top-selling malaria RDTs. Finally, it should be noted that some malaria RDT products, including some of these ‘top-selling’ ones, have been prequalified by the WHO Prequalification of Diagnostics Programme (WHO PQ) [27]; this has also possibly influenced procurement practice in some countries or by some procurers.

The proportion of countries procuring RDTs that did not meet the WHO recommendations was found to be higher in AMRO, SEARO and WPRO compared to other WHO regions. Interestingly, these ‘non-complying’ malaria RDT products were more often procured/sold through independent channels that are subject to fewer regulations. For instance, reporting of ‘non-complying’ products was much more frequent among countries purchasing RDTs with exclusive government funding (62%), compared to countries receiving funding from external donors (22%); of note, all self-funded countries reporting poor-complying products were from AMRO, SEARO or WPRO regions. Direct purchases between buyers and manufacturers represented around 21% of the procurement volumes, but accounted for more than half (54%) of the non-complying procured products. Importantly, sales transactions destined to the private sector comprised a higher proportion of RDTs not aligned with WHO criteria (32%), compared to public sector (5%). According to a manufacturer-specific market analysis conducted with the survey data (not disclosed here), private sector seems to attract a higher number of small/medium manufacturers, and especially those delivering products that do not comply or have not been submitted to PT evaluations. This is consistent with poor performance results reported from a small-scale study in 2009 among six malaria-endemic countries [20], and most probably due to the lack of regulation and/or less control of diagnostic products being used in the private health care sector in most malaria-endemic countries. A larger, multi-country survey conducted in 2011 [28] also documented that the overall number of products within the private sector was proportionally higher than in the public sector, thus supporting the finding of more diversified product assortment in the retail sector reported here. However, the performance of RDT products sold in the private sector could not be precisely evaluated in the aforementioned survey, since only the proportion of sales to the private sector (without product-specific identification) was collected; this should be the subject of a separate study.

Overall, African national authorities were those more familiar with the WHO–FIND programme and more frequently following the procurement recommendations. Furthermore, the large majority of non-complying products reported by AFRO NMCP/MoHs were Pan/*P. falciparum* RDTs that were actually ‘non-complying’ for performance of the ‘Pan’ test band only, which would only be relevant for the detection of *Plasmodium malariae* and *Plasmodium ovale* cases (*Plasmodium vivax* rarely existing in those regions), hence the most critical requirement of good performance for *P. falciparum* detection was still fulfilled.

### Coverage of the RDT market by LT

This study provides the first market-based estimation on the global coverage of the LT programme since its implementation in 2007. The analysis shows that 40% of sold lots and 70% of sold malaria RDTs were covered by the LT during the 2011–14 period. The difference among lot-based and RDT-based coverage estimations is due to bigger lot sizes of the product codes among the top sales, which also present higher LT coverage compared to the average. Manufacturers ensuring consistent high quality are those dominating the market, and are probably more capable of investing in the expansion of production plants (i.e., produce larger lots for ‘big’ customers). The reported trend towards increasing LT coverage of the RDT market correlates with the gradual introduction (initiated in 2011) of new policies requiring mandatory lot-testing for the recipients of major donor agencies. The survey has shown that all major procurers use the LT system and are mostly convinced about its usefulness and relevance. Only a few NMCP/MoHs expressed concerns about the shipment costs and transport-associated delays attributed to the LT submission process.

### Awareness and opinions about the programme

While the awareness on the WHO–FIND programmes was shown to be high among the surveyed stakeholders, some replies from NMCP/MoHs revealed limited knowledge of the programmes. This finding can be explained, among other reasons, by the quick turnover of NMCP coordinators observed in many countries, but also by a limited involvement of MoH staff in the RDT selection process (e.g., decisions directly taken by funding agency country offices). Reinforced communication about the programmes to this target group would further improve awareness at country level and contribute to national authorities taking ownership of quality assurance of malaria RDTs used in their country. When asking for opinions about the programmes, partial bias towards positive rating may have occurred since questionnaires were not anonymous. However, in the ‘Suggestions for improvement’ section, complaints and dislikes were openly expressed, so respondents’ feedback was generally perceived as honest.

## Limitations

The data collection procedures used in this study were subject to several limitations. First, some of the manufacturers did not report sales for all malaria RDTs manufactured by the company (some product codes were not included in their datasets). Second, private sector transaction figures are probably underestimated, as the inclusion criteria for the survey (i.e., ‘having submitted at least one product to PT evaluation’) probably left out several small companies that do not target public tenders. From the procurers’ side, only NMCP/MoHs and medium/big procurement organizations were invited to the survey, including some local procurers who had previously used the LT service, so private purchase requests (e.g., small country-based distributors, pharmacies, drug shops, private clinics) might be missing. It should also be noted that the survey respondents might have had different interpretations of the “public sector”, leading to some inconsistencies in their reporting. Whenever manufacturers had a related question, an explanation of what should be considered as the ‘public sector’ was provided. Third, another consequence of the above selection methods is that this produces some bias towards manufacturers and procurers who are already aware of the PT and LT programmes. However, given the fact that participation in the PT programme and use of the LT service are recommended by the WHO and even mandatory for Global Fund recipients, it is considered that these selection methods nevertheless allow including all major players in the malaria RDT market, which was the main purpose of this method. Fourth, as previously noted, the general estimations of manufacturers on their annual public sector sales ratio did not allow for distinction between products within the same company. Fifth, the occurrence of mistakes in the survey questionnaire’s self-reported data cannot be discounted, although large efforts were invested in detailed crosschecks to solve any detected discrepancies or omissions. And finally, the fact that this survey had some rate of non-response, that some manufacturers apparently did not report sales for some products (probably having been sold at small volumes), and that some small-size RDT manufacturers and local procurers are likely to be missing, affects to some extent the strength of the results reported here. Notably, small-volume transactions conducted by in-country, small size market actors, are probably underestimated, and the coverages of the PT and LT Programmes are probably slightly underestimated as well.

## Conclusions

The WHO–FIND Malaria RDT Evaluation programme has positively influenced procurement practices in the public sector between 2011–14. Malaria RDT products not in line with WHO recommendations seem to be mainly diverted to the private sector where capacity to enforce regulations and national policies is often insufficient, as well as to countries procuring with exclusive government funding and (probably) limited awareness of the PT programme. Advocacy and communication about the WHO–FIND programmes among country-based stakeholders, including those from the private healthcare sector, and more cooperation with national regulatory authorities could help increase compliance with WHO recommendations for procurement of malaria RDTs across all health sectors. In a collaborative implementation project in the private sector in five African countries, FIND and WHO have successfully contributed to the inclusion of specific malaria RDT quality assurance activities in the national policy documents and advocated strongly for a health sector-wide approach to implementation of these activities [29]. The survey reported here also revealed frequent concurrent use of different products and even product types in the same country and the same year, which highlights the importance of well-coordinated training to avoid the risk of confusion at the end user level.

While the submission of products to LT evaluation is consistently increasing, an estimated 30% of the sold RDTs (60% of lots) are not yet covered, so there is still room for expansion of the LT programme; again, an increased communication targeted at national-level stakeholders and regulatory authorities would be beneficial. Work is now underway, under funding from UNITAID, to transition to a more sustainable country-based LT model, already requested by various countries, with a more simple LT procedure based on recombinant antigen panels [30].

Overall, the survey demonstrates that both the PT and LT programmes are widely used and contribute to an increased quality of malaria RDTs sold and procured worldwide, with a large majority of stakeholders acknowledging their usefulness. The current ongoing work to transition both the PT and LT programmes to a more sustainable format is essential to ensure they can continue to play their important role.

## Additional files

**Additional file 1.** Questionnaire NMCP-MoH (FIND survey). Template of the survey questionnaire that was distributed among targeted NMCP/MoHs.

**Additional file 2.** Questionnaire procurers (FIND survey). Template of the survey questionnaire that was distributed among targeted RDT procurers.

**Additional file 3.** Questionnaire manufacturers (FIND survey). Template of the survey questionnaire that was distributed among targeted RDT manufacturers.

## Abbreviations

AFRO: WHO Regional Office for Africa
AMRO: WHO Regional Office for the Americas
EURO: WHO Regional Office for Europe
EMRO: WHO Regional Office for the Eastern Mediterranean
FIND: Foundation for Innovative New Diagnostics
IO: International Organization
LT: lot testing
MSF: *Médecins sans Frontières*
MoH: Ministry of Health
NGO: non-governmental organization
NMCP: National Malaria Control Programme
PQR: The Global Fund Price and Quality Reporting
PDS: panel detection score
PT: product testing
RDT: rapid diagnostic test
SEARO: WHO Regional Office for South-East Asia
UNICEF: United Nations Children’s Fund
US-PMI: US President’s Malaria Initiative
WHO: World Health Organization
WPRO: WHO Regional Office for the Western Pacific
