# Supplementary material for: Global survey of malaria rapid diagnostic test (RDT) sales, procurement and lot verification practices: assessing the use of the WHO–FIND Malaria RDT Evaluation Programme (2011–2014)
Source: Malar J. 2017 May 15;16:196. doi: 10.1186/s12936-017-1850-8 (PMC5433078; doi:10.1186/s12936-017-1850-8)
Supplement: Supplementary file 2 — Additional file 2. Questionnaire procurers (FIND survey). Template of the survey questionnaire that was distributed among targeted RDT procurers. [file 12936_2017_1850_MOESM2_ESM.doc]

**Impact of the WHO-FIND Malaria RDT Evaluation Programme:**

**Questionnaire for Malaria RDT Procurers**

Organization name:

Name and Position held in the organization:

*[NOTE: All responses in this questionnaire will be treated as strictly confidential. Data will be compiled in such a way that only aggregated figures will be reported or published. No product-specific information will be made available unless specified by the respondent.]*

**Part I:** Use and opinion of the Malaria RDT Evaluation Programme

|  |  |  |  | |  |  |  |  |
| --- | --- | --- | --- | --- | --- | --- | --- | --- |
| **1.** | Are you aware of the WHO-FIND Malaria RDT **Product Testing** Programme? | YES | |  |  | NO |  |  |
|  |  |  | |  |  |  |  |  |
|  |  |  | |  |  |  |  |  |
|  | • If YES, are you considering WHO-FIND Product Testing results for the **selection** of the malaria RDT **products** you procure? | YES | |  |  | NO |  |  |
|  |  |  | |  |  |  |  |  |

|  |  |  |  |  |  | |  | |  |
| --- | --- | --- | --- | --- | --- | --- | --- | --- | --- |
| **2.** | Are you aware of the WHO-FIND Malaria RDT **Lot Testing** Programme? | YES |  |  | NO | |  | |  |
|  |  |  |  |  |  |  |  |  |  |
|  |  |  |  |  |  | |  | |  |
|  | • If YES, do you use the Lot Testing Programme to **confirm the quality** of your RDT **lots** before distribution?  **check only if your organization sent the form to FIND requesting submission of a lot* | NO | | | |  | |  | |
|  |  | YES*-sometimes | | | |  | |  |  |
|  |  | YES*-always | | | |  | |  |  |
|  |  |  | | | |  | |  | |

|  |  |  |  |  |  |  |  |
| --- | --- | --- | --- | --- | --- | --- | --- |
| **3.** | Are the WHO-FIND Product Testing and/or Lot Testing Programmes mentioned in the **quality assurance policy / procurement criteria / tender documents** of your organization? | YES |  |  | NO |  |  |
|  |  |  |  |  |  |  |  |
|  |  |  |  |  |  |  |  |
|  | • If YES, could you provide the latest version of this document(s)? **[include attachment]** | YES |  |  | NO |  |  |
|  |  |  |  |  |  |  |  |

|  |  |  |  |  |  | |  |  |
| --- | --- | --- | --- | --- | --- | --- | --- | --- |
| **4.** | Regarding the Malaria RDT **Product Testing** programme:  *Please rate* ***between 0 - 10*** *(10 being the highest)* | | | | | | | |
|  | • In your opinion, does the programme have a **positive impact** on your procurement practices and the quality of products reaching the field? | | | | |  | |  |
|  |  | | | | | | | |
|  | • Does it **fulfill the needs** of your organization? | |  | | |  | |  |
|  |  | | | | | | | |

| **5.** | What **improvements** would you suggest for the Malaria RDT **Product Testing** Programme?  Please add any other **comment** (positive and/or negative) on the Programme you would like to share. |
| --- | --- |
|  |  |

|  |  |  |  |  |  | |  |  |
| --- | --- | --- | --- | --- | --- | --- | --- | --- |
| **6.** | Regarding the Malaria RDT **Lot Testing** programme:  *Please rate* ***between 0 - 10*** *(10 being the highest)* | | | | | | | |
|  | • In your opinion, does the programme have a **positive impact** on your procurement practices and the quality of products reaching the field? | | | | |  | |  |
|  |  | | | | | | | |
|  | • Does it **fulfill the needs** of your organization? | |  | | |  | |  |
|  |  | | | | | | | |

| **7.** | What **improvements** would you suggest for the Malaria RDT **Lot Testing** Programme?  Please add any other **comment** (positive and/or negative) on the Programme you would like to share. |
| --- | --- |
|  |  |

**Part II:** Malaria RDT procurement

| **8.** | What are the **main criteria** for RDT **product selection** in your organization?  *Please rate* ***between 0 – 10*** ***each*** *of these factors according to their relevance for decision-making*  *(10 being the highest):* | | | | | | |
| --- | --- | --- | --- | --- | --- | --- | --- |
|  | Price |  | National Regulatory Authority requirements | | |  |  |
|  | Lead-time (time delay for delivery) |  | Funding agency requirements | | |  |  |
|  | Storage conditions / Shelf-life |  | RDT evaluation in national labs | | |  |  |
|  | Ease of use / Training requirements |  | Field based evaluation of RDTs | | |  |  |
|  | WHO Product Testing performance |  | *Other:* |  |  |  |  |
|  |  |  |  | | |  |  |

| **9.** | Do you use the **“Interactive guide”** tool based on compiled Malaria RDT Product Testing Programme results (available at FIND’s website*) to select RDT products with specific characteristics suitable for your organization purposes? | NO |  |  |
| --- | --- | --- | --- | --- |
|  |  | YES-sometimes |  |  |
|  |  | YES-always |  |  |
|  |  | I don’t know this tool |  |  |
|  |  |  |  |  |

[* http://www.finddiagnostics.org/programs/malaria-afs/malaria/rdt_quality_control/product_testing/malaria-rdt-product-testing](http://www.finddiagnostics.org/programs/malaria-afs/malaria/rdt_quality_control/product_testing/malaria-rdt-product-testing/)/

| **10.** | Please provide the details below on the malaria RDT products your organization has **procured between 2011-2014**: | | | | | |
| --- | --- | --- | --- | --- | --- | --- |
|  | Product - Full Name | Catalogue Number | Country(s) | Year | Nº of RDTs  *[test units]* | Procured on behalf of…* |
|  |  |  |  |  |  |  |
|  |  |  |  |  |  |  |
|  |  |  |  |  |  |  |
|  |  |  |  |  |  |  |
|  |  |  |  |  |  |  |
|  |  |  |  |  |  |  |
|  |  |  |  |  |  |  |
|  |  |  |  |  |  |  |
|  |  |  |  |  |  |  |
|  |  |  |  |  |  |  |
|  |  |  |  |  |  |  |
|  |  |  |  |  |  |  |
|  |  |  |  |  |  |  |

*[add more rows if needed]*

** please specify if the purchase is done on behalf of another organization*

*NOTE: In case you preferred to provide this data in a different format, please contact* [*elisa.serra@finddiagnostics.org*](mailto:elisa.serra@finddiagnostics.org) *to check for alternative dataset table types that can be accepted.*

*🡪 Product-specific data in above table can be made publically available:*

***State Yes or No:* ……..…**

***- Thank you very much for your valuable contribution -***
