# Supplementary material for: Global survey of malaria rapid diagnostic test (RDT) sales, procurement and lot verification practices: assessing the use of the WHO–FIND Malaria RDT Evaluation Programme (2011–2014)
Source: Malar J. 2017 May 15;16:196. doi: 10.1186/s12936-017-1850-8 (PMC5433078; doi:10.1186/s12936-017-1850-8)
Supplement: Supplementary file 3 — Additional file 3. Questionnaire manufacturers (FIND survey). Template of the survey questionnaire that was distributed among targeted RDT manufacturers. [file 12936_2017_1850_MOESM3_ESM.doc]

**Impact of the WHO-FIND Malaria RDT Evaluation Programme:**

**Questionnaire for Manufacturers**

Company name:

Contact person and position:

*[NOTE: All responses in this questionnaire will be treated as strictly confidential. Data will be compiled in such a way that only aggregated figures will be reported or published. No product-specific nor company-specific information will be made available unless specified by the respondent.]*

**Part I:** Product Testing

|  |  |  |  |  |  |  | |  | |
| --- | --- | --- | --- | --- | --- | --- | --- | --- | --- |
| **1.** | *Please rate between 0 - 10 (being 10 the highest):* | | | | | | | | |
|  | • In your opinion, has the WHO-FIND Product Testing Programme helped you to **improve the quality** of your products? | | | | | |  | |  |
|  | • Does the WHO-FIND Product Testing Programme **fulfill the needs** of your company? | | | | | |  | |  |
| |  |  | |  |  |  | |  |  | |  | | --- | --- | --- | --- | --- | --- | --- | --- | --- | --- | --- | | **3.** | Does any of your **donors request** the use of Product Testing and/or  Lot Testing Programmes as a requirement? | | YES |  | | NO | |  | |  | |  |  | |  |  |  | |  |  | |  | |  |  | |  |  |  | |  |  | |  | |  | • If YES, please specify which donor and which is the requirement: |  | | | | | | |  | | |  |  | |  |  |  | |  |  | |  |  |  |  | |  |  |  | |  |  | |  | | --- | --- | --- | --- | --- | --- | --- | --- | --- | --- | --- | | **3.** | Does any of your **donors request** the use of Product Testing and/or  Lot Testing Programmes as a requirement? | | YES |  | | NO | |  | |  | |  |  | |  |  |  | |  |  | |  | |  |  | |  |  |  | |  |  | |  | |  | • If YES, please specify which donor and which is the requirement: |  | | | | | | |  | | |  |  | |  |  |  | |  |  | |  | |  | |  | | | |  | |  |

|  |  | | | | | | |
| --- | --- | --- | --- | --- | --- | --- | --- |
| **2.** | Has the Product Testing Programme **triggered any change** in your manufacturing process and/or quality control procedures? | | YES |  | NO |  |  |
|  |  | | | | | | |
|  |  | | | | | | |
|  | • If YES, please specify which ones: |  | | | | |  |
|  |  | | | | | | |

| **3.** | What **modifications** would you suggest for the WHO-FIND Product Testing Programme? Please add any other **comment** (positive and/or negative) on the Programme you would like to share. |
| --- | --- |
|  |  |

**Part II:** Lot Testing

|  |  |  | |  |  |  |  |  |
| --- | --- | --- | --- | --- | --- | --- | --- | --- |
| **4.** | Do you **submit** your **products** to the WHO-FIND Malaria RDT **Lot Testing** Programme before they are released in the market? **check only if your organization sent the form to FIND requesting submission of a lot* | YES*, always | | | | |  |  |
|  |  | YES*, but only when requested by the purchaser | | | | |  |  |
|  |  | NO | | | | |  |  |
|  |  | Other: |  | | | | |  |
|  |  |  | | | | |  |  |

|  |  |  | |  |  |  |  |  |
| --- | --- | --- | --- | --- | --- | --- | --- | --- |
| **5.** | Do you **inform** your **customers** about the option to submit the purchased RDTs to WHO-FIND Lot Testing evaluation? | | YES |  |  | NO |  |  |
|  |  |  | |  |  |  |  |  |

|  |  |  |  |  |  |  | |  | |
| --- | --- | --- | --- | --- | --- | --- | --- | --- | --- |
| **6.** | *Please rate between 0 - 10 (being 10 the highest):* | | | | | | | | |
|  | • In your opinion, has the WHO-FIND Lot Testing Programme helped you to **improve the quality** of your products? | | | | | |  | |  |
|  | • Does the WHO-FIND Lot Testing Programme **fulfill the needs** of your company? | | | | | |  | |  |
| |  |  | |  |  |  | |  |  | |  | | --- | --- | --- | --- | --- | --- | --- | --- | --- | --- | --- | | **3.** | Does any of your **donors request** the use of Product Testing and/or  Lot Testing Programmes as a requirement? | | YES |  | | NO | |  | |  | |  |  | |  |  |  | |  |  | |  | |  |  | |  |  |  | |  |  | |  | |  | • If YES, please specify which donor and which is the requirement: |  | | | | | | |  | | |  |  | |  |  |  | |  |  | |  |  |  |  | |  |  |  | |  |  | |  | | --- | --- | --- | --- | --- | --- | --- | --- | --- | --- | --- | | **3.** | Does any of your **donors request** the use of Product Testing and/or  Lot Testing Programmes as a requirement? | | YES |  | | NO | |  | |  | |  |  | |  |  |  | |  |  | |  | |  |  | |  |  |  | |  |  | |  | |  | • If YES, please specify which donor and which is the requirement: |  | | | | | | |  | | |  |  | |  |  |  | |  |  | |  | |  | |  | | | |  | |  |

|  |  | | | | | | |
| --- | --- | --- | --- | --- | --- | --- | --- |
| **7.** | Has the Lot Testing Programme **triggered any change** in your manufacturing process and/or quality control procedures? | | YES |  | NO |  |  |
|  |  | | | | | | |
|  |  | | | | | | |
|  | • If YES, please specify which ones: |  | | | | |  |
|  |  | | | | | | |

| **8.** | What **modifications** would you suggest for the WHO-FIND Lot Testing Programme? Please add any other **comment** (positive and/or negative) on the Programme you would like to share. |
| --- | --- |
|  |  |

** Additional data (if possible to share it, under strict confidential cover):*

| **9.** | Could you provide a brief description of your malaria RDT **internal Quality Control** and **lot-release testing** procedures, for the device, buffer and accessories? *(type/origin of samples, at what concentration/density, Nº of RDTs tested per lot, criteria for accepting/rejecting, etc.)* |
| --- | --- |
|  |  |

**Part III:** Malaria RDT sales / deliveries

| **10.** | From years 2011 to 2014, how many **RDTs** and how many **RDT lots** have been released per product? | | | | | | | | | |
| --- | --- | --- | --- | --- | --- | --- | --- | --- | --- | --- |
|  | Product | | 2011 | | 2012 | | 2013 | | 2014 | |
|  | Product Name | Catalogue Number | Nº RDTs | Nº Lots | Nº RDTs | Nº Lots | Nº RDTs | Nº Lots | Nº RDTs | Nº Lots |
|  |  |  |  |  |  |  |  |  |  |  |
|  |  |  |  |  |  |  |  |  |  |  |
|  |  |  |  |  |  |  |  |  |  |  |
|  |  |  |  |  |  |  |  |  |  |  |
|  |  |  |  |  |  |  |  |  |  |  |
|  |  |  |  |  |  |  |  |  |  |  |

*[add more rows if needed]*

*NOTE: In case you preferred to provide this data in a different format, please contact* [*elisa.serra@finddiagnostics.org*](mailto:elisa.serra@finddiagnostics.org) *to check for alternative dataset table types that can be accepted.*

*🡪 Product-specific data in above table can be made publically available:*

***State Yes or No: …………***

| **11.** | Which proportion of the malaria RDT sales were destined to the **Public Sector**? *(provide % estimation)* | 2011 | 2012 | 2013 | 2014 |
| --- | --- | --- | --- | --- | --- |
|  |  |  |  |  |

| **12.** | What is the average **size** (Nº of units) of the malaria RDT **lots** you manufacture? | mean | *minimum* | *maximum* |
| --- | --- | --- | --- | --- |
|  |  |  |

| **13.** | In which **countries** have your malaria RDTs been delivered between 2011-2014? |
| --- | --- |
|  |  |

***- Thank you very much for your valuable contribution -***
